# Inhibition of HCN Channels Enhances Oxidative Stress and Autophagy of NRK-52E Cells Under NH_4_Cl Treatment

**DOI:** 10.3390/ijms26189227

**Published:** 2025-09-21

**Authors:** Zinaeli López-González, Laura I. Escobar, Daniel León-Aparicio, Abirán Fernando Mejía-Peralta, Teresa Padilla-Flores, Isabel Larre, Carolina Salvador, Omar Noel Medina-Campos, José Pedraza-Chaverri, Marisol de la Fuente-Granada

**Affiliations:** 1 Departamento de Fisiología, Facultad de Medicina, Universidad Nacional Autónoma de México (UNAM), Mexico City 04510, Mexico; zlopez@facmed.unam.mx (Z.L.-G.); dleon@facmed.unam.mx (D.L.-A.);; 2 Departamento de Biología, Facultad de Química, Universidad Nacional Autónoma de México (UNAM), Mexico City 04510, Mexicopedraza@unam.mx (J.P.-C.); 3 Departamento Medicina Genómica y Toxicología Ambiental, Instituto de Investigaciones Biomédicas, Universidad Nacional Autónoma de México (UNAM), Mexico City 04510, Mexico; mdelafuente@iibiomedicas.unam.mx

**Keywords:** Hyperpolarization-activated cyclic nucleotide-gated cation (HCN) channels, autophagy, oxidative stress, ZD7288, HCN3 lysosomal channel, HCN3 mitochondrial channel

## Abstract

The hyperpolarization-activated cyclic nucleotide-gated (HCN) channels in the kidney participate in reabsorbing potassium (K^+^) and ammonium (NH_4_^+^) in the nephron, contributing to the acid–base balance. Acidosis is a metabolic condition of renal tubular acidosis and chronic kidney disease. Acidosis stimulates the production of mitochondrial reactive oxygen species (mROS), activating protective mechanisms dependent on mitochondrial membrane potential (Δψm) such as autophagy. The HCN3 channel is expressed in the plasma membrane, mitochondria (mitoHCN3), and lysosomes (lysoHCN3) of the rat proximal tubule. In this work we aimed to investigate the role of HCN3 in autophagy, mROS production, and Δψm in cultured rat proximal tubule cells (NRK-52E) exposed to ammonium chloride (NH_4_Cl). NH_4_Cl arrested autophagic flux and produced extracellular acidosis and, under this condition, mitoHCN3 and lysoHCN3 were up-regulated. NH_4_Cl or/and ZD7288, a specific blocker of HCN channels, enhanced mROS. ZD7288 in NH_4_Cl conditions at 24 h stimulated autophagy by reducing Beclin1, LC3BII, p62, and Parkin in an mROS- or Δψm independent pathway. Therefore, ZD7288 reverted NH_4_Cl inhibited autophagy through lysoHCN3 inhibition. Oxidative stress induced by H_2_O_2_ up-regulated mitoHCN3 expression, while Tiron had the opposite effect. In conclusion, inhibition of mito- and lysoHCN3 channels by ZD7288 can protect against mitochondrial oxidative stress and stimulate the lysosome–autophagy pathway in response to NH_4_Cl treatment.

## 1. Introduction

The lungs and kidneys keep arterial pH within a narrow range of 7.35 to 7.46. The kidney regulates plasma bicarbonate concentration by excreting hydrogen ions as ammonium (NH_3_/NH_4_^+^) and nonvolatile acids [1]. Metabolic acidosis frequently appears as a clinical symptom in patients suffering from chronic kidney disease (CKD) and renal tubular acidosis (RTA). Acidosis triggers oxidative stress provoking inflammation and fibrosis, worsening the damage in the kidney [2]. During acidosis, the liver releases glutamine which is captured by the kidney and catabolized in the mitochondria of the proximal tubule of the nephron. Glutamine catabolism produces ammonium and bicarbonate facilitating urinary acid excretion and acid–base balance by reabsorbing bicarbonate [3].

The proximal tubule in the kidney is responsible for reabsorbing most of the sodium and bicarbonate in the body, spending a high amount of ATP to facilitate the transcellular movement of various nutrients such as amino acids, glucose, and important anions such as phosphate and citrate, among other electrolytes. For this reason, this first segment of the nephron contains the highest number of mitochondria and performs their selective degradation by autophagy (mitophagy). Maintenance of mitochondrial homeostasis is vital for kidney function. Mitophagy acts as a quality control process in which impaired mitochondria are enclosed within autophagosomes and autolysosomes for degradation, allowing cells to cope with stressful conditions such as oxidative stress caused by metabolic acidosis [4,5].

During acidosis mitochondria reduce their oxidative phosphorylation and Ca^2+^ accumulation capacity, favoring the production of reactive oxygen species (mROS) in the kidney [5,6]. A disturbance in mROS homeostasis has been associated with essential hypertension, cardiovascular disorders, neurodegenerative diseases, chronic obstructive pulmonary diseases, CKD, and cancer [7,8]. Mitochondria are the primary internal source of ROS, rendering them highly susceptible to harm during physiological adaptations and challenging conditions [9]. Paradoxically, a moderate increase in mROS levels activates mitophagy, nevertheless, an overproduction can lead to pronounced mitochondrial membrane depolarization and apoptosis, if not effectively countered by mitophagy [9] or antioxidant defenses [10].

The hyperpolarization-activated and cyclic nucleotide-gated (HCN) cation channels are activated at hyperpolarizing voltages of −60 to −100 mV [11,12]. In the kidney HCN channels contribute to the reabsorption and recycling of K^+^/NH_4_^+^, playing an important role in maintaining acid–base homeostasis [13,14]. Mitochondrial potassium HCN channels (mitoHCN) regulate the mitochondrial membrane potential (Δψm), oxygen consumption, and ATP synthesis in the kidney [15] and the heart [16]. We previously found that rats under metabolic acidosis overexpressed HCN3 in lysosomal membranes (lysoHCN3) without changes to HCN3’s relative abundance in brush border membranes and mitochondria from cortical proximal tubule cells [17].

Block of HCN channels with ivabradine (Iva) or its analog ZD7288, shows cardio-, neuro-, and/or renal protective effects. Iva reduces oxidative injury and endothelial dysfunction in cardiac ischemia [18] and promotes autophagy by blocking the PI3K/AKT/mTOR/p7056K pathway during myocardial infarction [19]. In the kidney, Iva improves the renal function of patients with sepsis and secondary renal dysfunction and reduces oxidative stress during ischemia/reperfusion (I/R) injury [20]. On the other hand, ZD7288 exhibits neuroprotection by accelerating autophagic degradation in hippocampal cells and attenuating apoptosis in a model of cerebral ischemia–reperfusion [21,22].

In this work we aimed to evaluate the role of mitoHCN3 and lysoHCN3 channels in autophagy, mROS production, and Δψm through their inhibition with ZD7288 in cultured rat proximal tubule cells (NRK-52E) exposed to NH_4_Cl.

## 2. Results

### 2.1. NH_4_Cl Causes Extracellular Acidosis and Autophagy Inhibition

**Figure 1:**
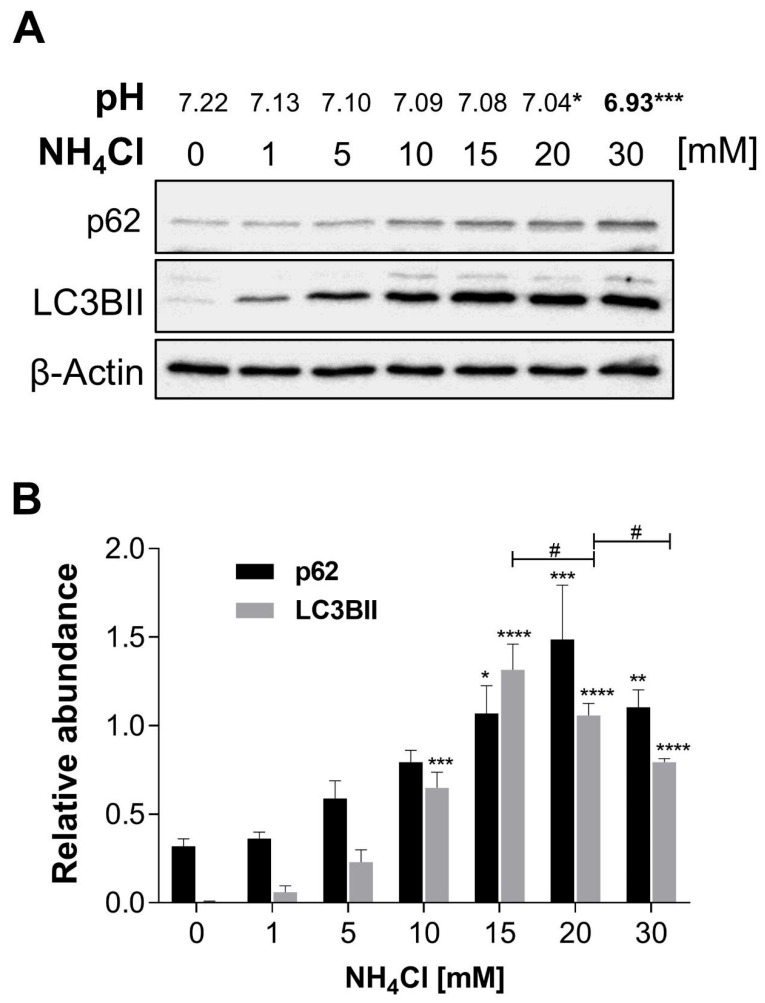
NH_4_Cl produces extracellular acidosis and arrest of the autophagic flux. (**A**) pH dependence of NH_4_Cl concentration. Immunoblots of p62 and LC3BII from total homogenates of NRK-52E cells exposed to variable concentrations of NH_4_Cl for 24 h. (**B**) Densitometric analysis shows the relative abundance of p62 and LC3BII, normalized to β-Actin (loading control). All data are represented as the mean ± SEM (*n* = 3). Ordinary one-way ANOVA, * compared to the control group (Dunnett’s post hoc test), * *p* < 0.05, ** *p* < 0.01, *** *p* < 0.001, **** *p* < 0.0001; ^#^ comparison between preselected groups (Sidak’s post hoc test), ^#^ *p* < 0.05.

Extracellular acidosis was established with NH_4_Cl 30 mM (pH 6.93 ± 0.070 vs. pH 7.22 ± 0.007; Figure 1) without altering apoptosis or necrosis of NRK-52E cells (Figure S1). NH_4_Cl provoked LC3BII and p62 accumulation compared to control (0.006 ± 0.003 vs. 1.32 ± 0.15 and 0.32 ± 0.041 vs. 1.49 ± 0.31, respectively) (Figure 1).

### 2.2. NH_4_Cl Inhibits mitoHCN3 Proteolysis and Increases lysoHCN3 Expression

**Figure 2:**
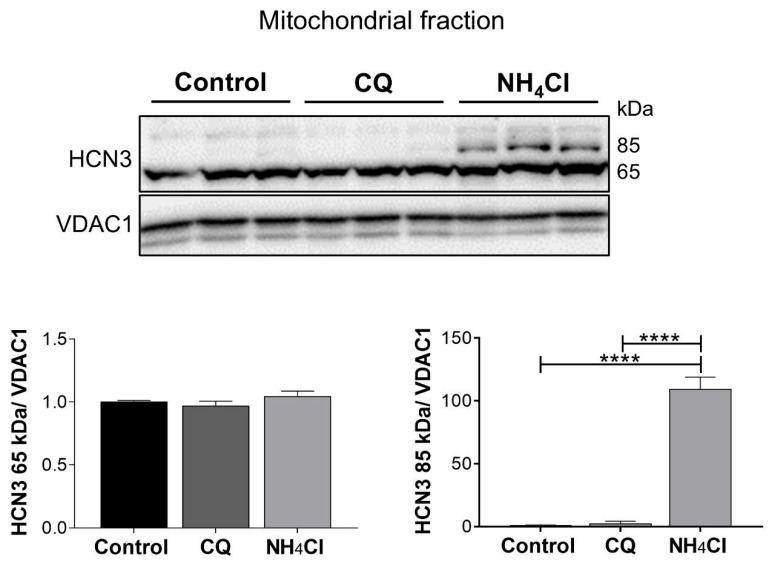
Identification of mitoHCN3 in NRK-52E cells and its regulation by chloroquine (CQ) and NH_4_Cl-induced acidosis. Full-length (85 kDa) and N-terminal truncated (65 kDa) mitoHCN3 isoforms were immunodetected with an antibody targeting a C-terminal epitope in mitochondrial fractions of NRK-52E cells, control and exposed to NH_4_Cl (30 mM) and CQ (50 μM) for 24 h. Graphs display the relative abundance of mitoHCN3, normalized to VDAC1 (loading control) and the control group. Data shown in triplicate are represented graphically as the mean ± SEM. Ordinary one-way ANOVA, followed by Newman–Keuls multiple comparisons post hoc test, **** *p* < 0.0001.

**Figure 3:**
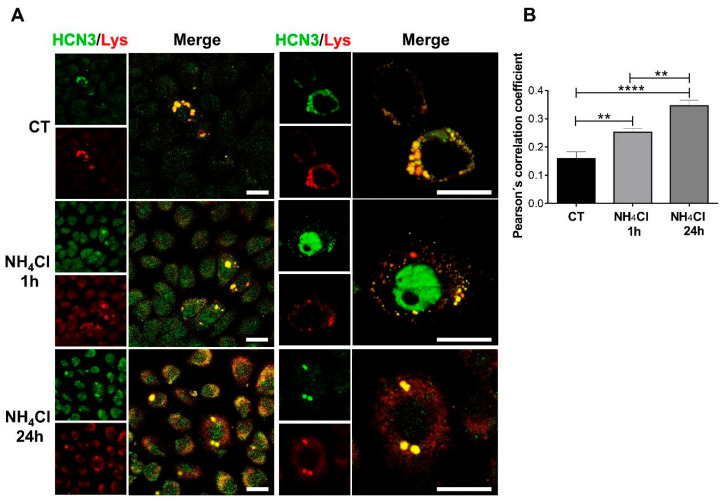
NH_4_Cl promotes time-dependent HCN3 expression in lysosomes (LysoHCN3). (**A**) Representative confocal images of NRK-52E cells in absence (CT) or presence of NH_4_Cl (1 and 24 h) with colabeling HCN3 channels (green) and lysosomes (Lys, red). Scale bar 20 μm. (**B**) Quantitative analysis of colocalization using Person’s coefficient. Data are represented as the mean ± SEM (*n* = 10 cells). Ordinary one-way ANOVA, followed by Newman–Keuls multiple comparisons post hoc test, ** *p* < 0.01, **** *p* < 0.0001.

The N-terminal truncated HCN3 isoform (65 kDa) predominates in mitochondria of NRK-52E cells (Figure 2), as we previously found in cardiomyocytes [16]. As a control, a comparative analysis with chloroquine (CQ), a common autophagy inhibitor, was performed. The full-length mitoHCN3 isoform (85 kDa) was expressed in NH_4_Cl in contrast to CQ treatment (Figure 2). Like what we observed in kidney tissue, expression of HCN3 channels in lysosomes (lysoHCN3) of NRK-52E cells was confirmed together with its time-dependent up-regulation by NH_4_Cl (Figure 3).

### 2.3. Time-Dependent Effect of ZD7288 on Mitophagy

**Figure 4:**
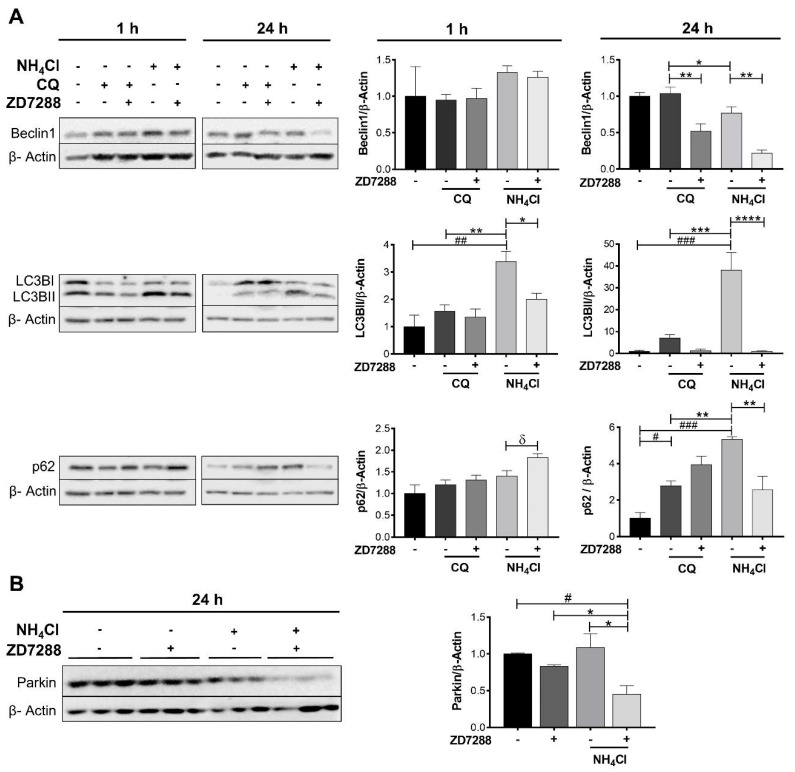
Effect of ZD7288 on mitophagy with chloroquine (CQ) or NH_4_Cl-induced acidosis after 1 and 24 h. (**A**) Immunoblotting of Beclin1, LC3B, p62, and (**B**) Parkin from total homogenates of NRK-52E cells exposed to ZD7288 (50 µM) for 1 h or 24 h. Graphs show the densitometric analysis normalized to β-Actin (loading control) and the control group. Data are represented as the mean ± SEM (*n* = 3). Ordinary one-way ANOVA, ^#^ compared to the control group (Dunnett’s post hoc test) ^#^ *p* < 0.05, ^##^ *p* < 0.01, ^###^ *p* < 0.001; * comparison between preselected groups (Sidak’s post hoc test) * *p* < 0.05, ** *p* < 0.01, *** *p* < 0.001, **** *p* < 0.0001; ^***δ***^ Unpaired Student’s *t*-test, ^***δ***^ *p*< 0.05.

Beclin1, LC3BII, p62, and Parkin were quantified under CQ or NH_4_Cl treatment at 1 and 24 h (Figure 4). ZD7288 plus NH_4_Cl for 1 h did not change Beclin1 but decreased LC3BII (3.4 ± 0.4 vs. 2.0 ± 0.2) and increased p62 (1.4 ± 0.1 vs. 1.8 ± 0.1), suggesting an early blockage of mitophagy. After 24 h, the effect of NH_4_Cl was higher on LC3BII than p62 levels (1.0 ± 0.5 vs. 38.2 ± 7.8 and 1.0 ± 0.3 vs. 5.3 ± 0.1, respectively) compared to CQ (1.0 ± 0.5 vs. 7.1 ± 1.5 and 1.0 ± 0.3 vs. 2.8 ± 0.3, respectively). ZD7288 plus CQ provoked a small down-regulation on Beclin1 (1.0 ± 0.1 vs. 0.5 ± 0.1) and then a mild activation of autophagy. In contrast, ZD7288 plus NH_4_Cl accelerated mitophagy supported by lower levels of all the biomarkers: Beclin1 (0.77 ± 0.08 vs. 0.22 ± 0.05), LC3BII (38.2 ± 7.8 vs. 0.22 ± 0.05), p62 (5.3 ± 0.1 vs. 2.5 ± 0.7), and Parkin (1.1 ± 0.1 vs. 0.4 ± 0.1) (Figure 4).

**Figure 5:**
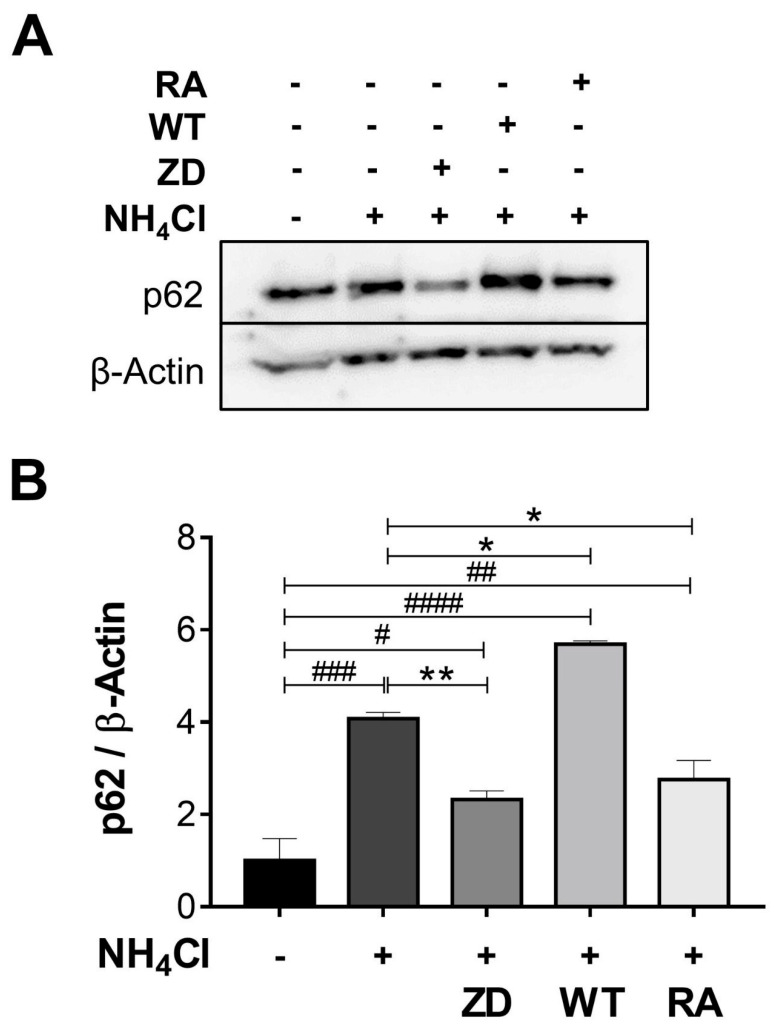
Effect of ZD7288 (ZD), Wortmannin (WT), and Rapamycin (RA) on autophagy. (**A**) Immunoblotting of p62 from total homogenates of NRK-52E cells subjected to NH_4_Cl-induced acidosis and ZD (50 µM), WT (100 nM), or RA (200 nM) for 24 h. (**B**) The bar graph shows the relative abundance of p62, normalized to β-Actin (loading control) and the control group. Data are represented as the mean ± SEM (*n* = 3). Ordinary one-way ANOVA, ^#^ compared to the control group (Dunnett’s post hoc test) ^#^ *p* < 0.05, ^##^ *p* < 0.01, ^###^ *p* < 0.001, ^####^ *p* < 0.0001; * comparison between preselected groups (Sidak’s post hoc test) * *p* < 0.05, ** *p* < 0.01.

To corroborate the effect of ZD7288 on autophagy, a comparative assay with wortmannin (WT) and rapamycin (RA) was performed. As expected, NH_4_Cl provoked accumulation of p62 (1.0 ± 0.14 vs. 4.0 ± 0.1) while ZD7288 caused the opposite effect (4.0 ± 0.1 vs. 2.3 ± 0.2) after 24 h. Accordingly, WT up-regulated (4.0 ± 0.1 vs. 5.7 ± 0.07) while rapamycin down-regulated (4.0 ± 0.1 vs. 2.7 ± 0.4) p62. Therefore, ZD7288 was a potent inducer of autophagy under NH_4_Cl treatment (Figure 5).

### 2.4. ZD7288 and/or NH_4_Cl Increases Oxidative Stress

**Figure 6:**
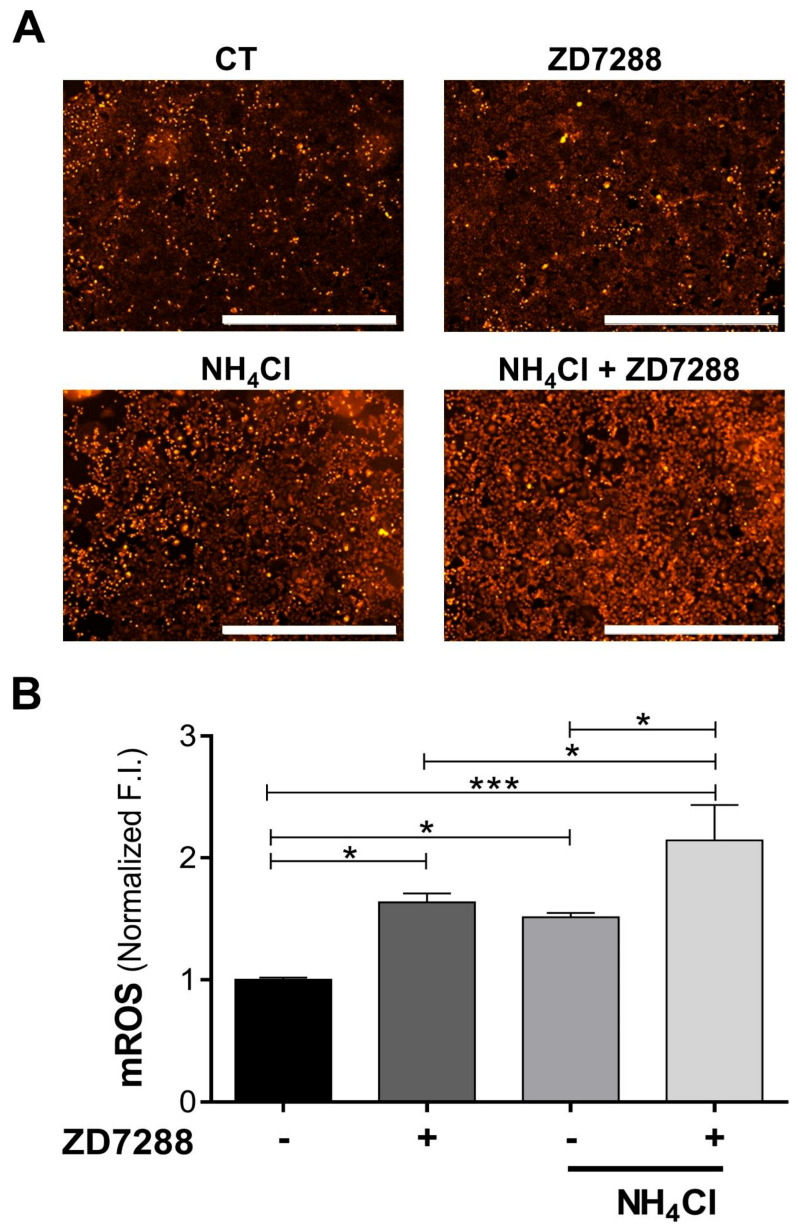
NH_4_Cl-induced acidosis and ZD7288 stimulate mROS production. (**A**) Representative images of mitoSOX-loaded NRK-52E cells under basal (CT), NH_4_Cl (30 mM), and ZD7288 (50 μM) conditions for 24 h. Scale bar 1 mm. (**B**) Bar graph displays the fluorescence intensity (F.I.) analysis. Data are represented as the mean ± SEM (*n* = 5). Ordinary one-way ANOVA, followed by Newman– Keuls multiple comparisons post hoc test, * *p* < 0.05, *** *p* < 0.001.

ZD7288 (1.0 ± 0.02 vs. 1.64 ± 0.07) or NH_4_Cl (1.0 ± 0.02 vs. 1.51 ± 0.03) stimulated mROS production and together doubled it (1.0 ± 0.02 vs. 2.14 ± 0.29; Figure 6).

**Figure 7:**
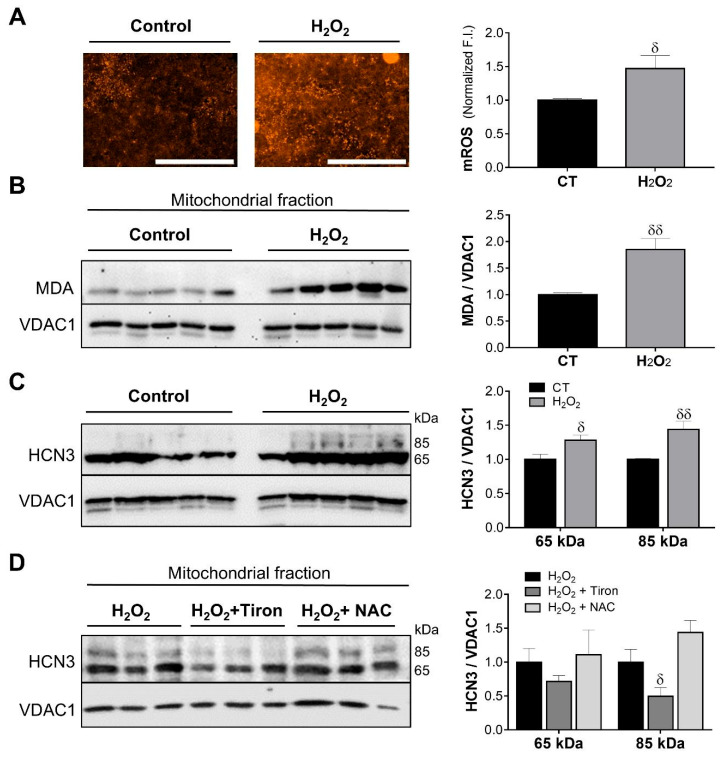
mitoHCN3 is up-regulated by H_2_O_2_-induced oxidative stress. (**A**) Representative images of mitoSOX-loaded NRK-52E cells under control (CT) and oxidative stress (H_2_O_2_) conditions (*n* = 3). Scale bar 1 mm. (**B**) Immunoblots show the effect of oxidative stress on mitochondrial expression of malondialdehyde (MDA) and (**C**) mitoHCN3 (*n* = 5). (**D**) Tiron decreased mitoHCN3 abundance (full-length, 85 kDa) under oxidative stress (*n* = 3). Graphs display the quantification of fluorescence intensity (F.I.) or optical density normalized to VDAC1, used as loading control for the mitochondrial fraction. Data are represented as the mean ± SEM. Unpaired Student’s *t*-test, ^***δ***^ *p* < 0.05; ^***δδ***^ *p* < 0.01.

H_2_O_2_-induced oxidative stress increased mROS (1.0 ± 0.02 vs. 1.47 ± 0.19; Figure 7A) and malondialdehyde (MDA) levels (1.0 ± 0.03 vs. 1.85 ± 0.21; Figure 7B). H_2_O_2_ up-regulated the full-length (1.0 ± 0.01 vs. 1.44 ± 0.12) and the N-terminal truncated (1.0 ± 0.08 vs. 1.28 ± 0.08) HCN3 isoforms in mitochondria (Figure 7C). This effect was confirmed with Tiron and N-acetylcysteine (NAC) in cells treated simultaneously with H_2_O_2_. Unlike NAC, Tiron decreased the abundance of the full-length mitoHCN3 (1.0 ± 0.18 vs. 0.49 ± 0.12; Figure 7D). Therefore, oxidative stress stimulated either by NH_4_Cl or H_2_O_2_ up-regulates HCN3 isoforms in mitochondria.

### 2.5. Mitophagy Stimulated by ZD7288 in NH_4_Cl Is Independent of ROS and Δψm

**Figure 8:**
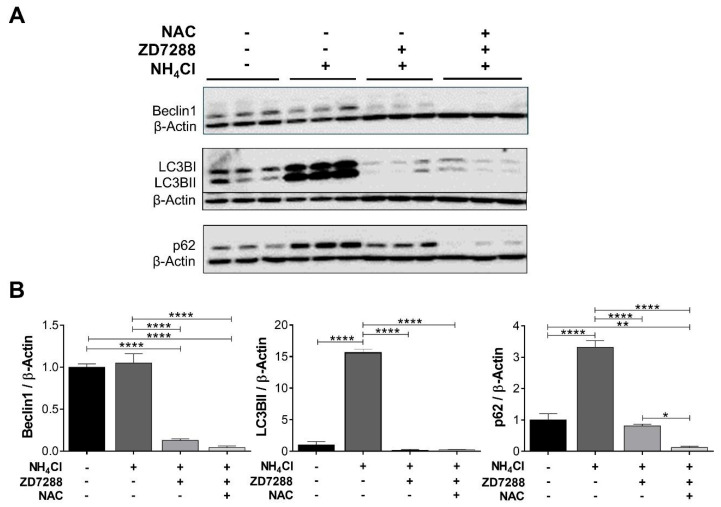
ZD7288-induced autophagy in acidosis is mediated by a ROS-independent pathway. (**A**) Immunoblotting of Beclin1, LC3B, and p62 from total homogenates of NRK-52E cells subjected to NH_4_Cl-induced acidosis, ZD7288 (50 µM), and N-acetylcysteine (NAC; 10 mM) for 24 h. (**B**) Graphs show the densitometric analysis normalized to β-Actin (loading control) and the control group. Data shown in triplicate are represented graphically as the mean ± SEM. Ordinary one-way ANOVA, followed by Newman–Keuls multiple comparisons post hoc test, * *p* < 0.05, ** *p* < 0.01, **** *p* < 0.0001.

Since moderate levels of mROS and mitochondrial depolarization can trigger mitophagy [23], we examined the effect of NAC plus ZD7288 in acidosis on Beclin1, LC3BII, and p62. According to our previous results, ZD7288 activated autophagy after 24 h. Interestingly, NAC did not reverse this effect (Figure 8), demonstrating a ROS-independent autophagy. Reduced p62 levels by NAC suggests a transcriptional effect rather than an increase in the rate of p62 degradation [24].

**Figure 9:**
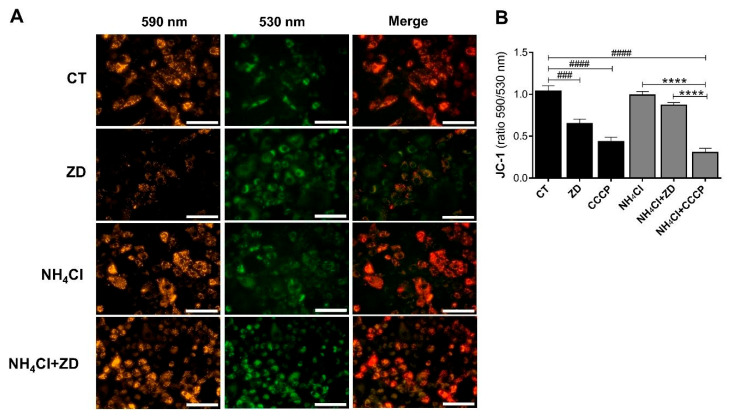
ZD7288-induced autophagy in acidosis is mediated by a Δψm-independent pathway. (**A**) Representative images of JC-1 signal in NRK-52E cells exposed to ZD7288 (50 μM, ZD) under basal conditions (CT) and NH4Cl-induced acidosis for 24 h. Scale bar 100 μm. (**B**) The graph shows JC-1 analysis represented as the ratio of fluorescence at 590 nm (hyperpolarization) and 530 nm (depolarization) normalized to the CT group (*n* = 5). CCCP (50 μM, 10 min) was used as a control of the uncoupling effect. Data are represented as the mean ± SEM. Ordinary one-way ANOVA, ^#^ compared to the control group (Dunnett’s post hoc test), ^###^ *p* < 0.001, ^####^ *p* < 0.0001; * comparison between preselected groups (Sidak’s post hoc test), **** *p* < 0.0001.

We evaluated the effect of ZD7288 on Δψm in basal and NH_4_Cl conditions after 24 h. ZD7288 had an uncoupling effect like CCCP in basal conditions (Figure 9). However, depolarization caused by ZD7288 was not sufficient to induce cell apoptosis or necrosis (Figure S1). Remarkably, Δψm did not change in NH_4_Cl, nor when ZD7288 was added (Figure 9A,B). These results confirmed that mitophagy stimulated by ZD7288 under NH_4_Cl treatment cannot be mediated by Δψm.

## 3. Discussion

Metabolic acidosis (MA) alters mitochondrial metabolism of the proximal tubule because of increased glutamine catabolism [25]. MA provokes in the mitochondria lower efficiency of oxidative phosphorylation, Ca^2+^ accumulation, and ROS production, which represents a significant stress [6]. MA induces autophagy in proximal tubular cells as an adaptive mechanism to maintain the mitochondrial functions and the proper excretion of NH_4_Cl in the urine to restore acid–base balance [5].

Hereditary distal renal tubular acidosis (dRTA) consists of impairment of apical H^+^ secretion or basolateral bicarbonate reabsorption, produced by mutations in genes encoding V-ATPase or the anion exchanger Cl^-^/HCO_3_^−^ (kAE1). dRTA is the cause of decreased ammonium (NH_4_^+^) excretion and, in consequence, impaired urine acidification, leading to simultaneous MA, hypokalemia, hypercalciuria, hypocitraturia, and nephrocalcinosis [1,26]. Although alkaline therapy can ameliorate several of dRTA symptoms, hearing loss is progressive and irreversible. In a zebrafish model pH imbalance due to V-ATPase deficiency provoked systemic acidosis and hair cell degeneration in the inner ear, due to autophagy inhibition despite an alkaline treatment [27].

Blockade of HCN channels develop cardio-, neuro-, and reno-protective effects by increasing autophagy or reducing cellular apoptosis [28]. However, it is still uncertain if these protective effects could be associated with mitochondrial [15] and/or lysosomal [17] HCN channels. In this work we studied the role of mitoHCN3 and lysoHCN3 in autophagy, mROS production, and Δψm changes under basal and NH_4_Cl conditions in NRK-52E cells.

In the kidney glutamine is converted to the buffer NH_4_^+^/NH_3_ in the proximal tubule to eliminate the body’s acid load and maintain an acidic urinary pH of 5–6. NH_4_Cl has been used as an acidifying agent in humans and experimental animals [29,30,31], as well as in in vitro models. Numerous studies report the use of NH_4_Cl in cell cultures: primary cultures of mouse cerebral astrocytes [32], myocytes of chickens [33] and rabbits [34], hippocampal neurons [35], as well as in epithelial cells from the thick ascending limb of Henle’s loop of the rabbit kidney [36] and NRK-52E cells [17].

Incubation with 30 mM NH_4_Cl for 24 h produced extracellular acidosis (pH 6.93) regarding a control pH of 7.2. This pH is consistent with previous reports, which demonstrate that PCO_2_ of the proximal tubule fluid of the rat renal cortex (60 mm Hg, pH 7.2) is more acidic than arterial pH (40 mm Hg; pH 7.4) [37]. We also induced extracellular acidosis with HCl. However, this approach resulted in unstable pH conditions, as CO_2_ in the incubator caused fluctuations that were difficult to control over a 24 h treatment period. In contrast, the intrinsic buffering capacity of NH_4_Cl allowed us to maintain a stable acidic pH throughout the assay.

Renal hypertrophy developed in acidosis has been associated with increased NH_4_Cl production rather than acidosis [38]. The mechanism by which NH_4_Cl inhibits protein degradation is attributed to lysosomal alkalinization [39,40], in agreement with the accumulation of LC3BII and p62 observed in this work.

As we previously found in the kidney and the heart mitochondria, we detected mostly the N-terminal truncated HCN3 isoform (65 kDa) in mitochondria of NRK-52E cells, generated by the N-terminal proteolysis at the residues Ala162–Ile163 [41]. The full-length HCN3 isoform (85 kDa) observed with NH_4_Cl treatment suggests its proteolysis by an endogenous protease in basal conditions which is inhibited by acidosis or oxidative stress. HCN channels present putative cleavage sites in their polypeptide sequences for membrane-bound metalloendopeptidases [41]. In this regard, we previously found HCN3 colocalization with the metalloendopeptidase neprilysin in the apical membrane of the rat straight proximal tubule S3 [14].

Previously, we identified lysoHCN3 channels in proximal tubule cells and its up-regulation in acidotic rats [17]. In this work, we confirmed lysoHCN3 expression and its up-regulation by NH_4_Cl in NRK-52E cells.

Autophagy inhibitors such as chloroquine (CQ), bafilomycin A1, pepstatin A, and E64d are commonly used to study autophagy [42]. Since NH_4_Cl inhibits the autophagic flux [43] we compared its effects with CQ. LC3BII and p62 were accumulated more with NH_4_Cl than CQ after 24 h, indicating a greater recruitment of the autophagic protein machinery in NH_4_Cl. This is supported by findings demonstrating that acidosis in vivo and in vitro promotes mitophagy to maintain proper mitochondrial functions, favoring the increase in LC3BII-tagged autophagosomes in the proximal tubule [5]. In contrast to other inhibitors, NH_4_Cl promotes the recruitment of the autophagic machinery through inhibition of the PI3K/Akt/mTOR signaling pathway [44].

Our findings demonstrate that ZD7288-induced autophagy in NH_4_Cl occurs by a pathway independent of ROS and Δψm. We hypothesized that blockade of lysoHCN3 channels by ZD7288 in NH_4_Cl-inhibited autophagy would prevent NH_4_^+^ uptake by lysosomes and then their alkalinization [39,40], favoring autolysosome formation. Therefore, lysoHCN3 appears as a key player in lysosomal acid–base balance regulated by glutamine catabolism in the kidney [39,43]. In agreement with this hypothesis, Iva and ZD7288 enhance the autophagic degradation of cardiac and hippocampal cells via an augmented fusion of autophagosomes and lysosomes [19,21].

ZD7288 plus NH_4_Cl after 1 h down-regulated LC3BII and up-regulated p62, supporting a time-dependent inhibition of autophagy [19]. Early blockage of autophagy by ZD7288 reveals the importance of the uncoupling effect of mitoHCN3 on autophagy.

Previously, we observed that HCN3 overexpression in HEK293 did not change the respiratory control index but instead decreased mitochondrial ATP production, most likely because of mitophagy [15]. Accordingly, overexpression of another mitoHCN (HCN4) in HEK293 cells caused higher LC3BII levels than the control (Figure S2).

ZD7288 increased mROS levels without developing apoptosis. In this context, the relationship between mROS production and mitoK channels has been documented in cancer. Blockade of the mitoKv1.3 channel increased ROS levels, triggering apoptosis in glioblastoma and melanoma cells [45,46]. An increase in mROS occurs in the absence of the BKCa mitoK channel in glioblastoma cells [47]. Likewise, inhibition of mitoCa^2+^-activated K^+^ channels of intermediate conductance (IKCa) leads to an elevation of ROS and disruption of the mitochondrial network in cell lines of melanoma, pancreatic ductal adenocarcinoma, and breast cancer [48]. Mitochondrial dynamics linked to ROS production have been documented with the mitochondrial potassium channel KCa3.1, a calcium-activated potassium channel expressed in renal proximal tubular cells. TGF-β1 increases mitochondrial ROS and fission and suppresses fusion, which is reversed by blockade or lack of KCa3.1 in HK2 cells [49]. Further studies should be performed to elucidate the effect of ZD7288 on mitochondrial dynamics.

Our results confirm the strong dependence of mROS generation on Δψm [50,51]. Uncoupling proteins create a regulatory feedback mechanism to lower the electrochemical proton gradient (Δp) and diminish mROS generation [52]. Indeed, uncouplers of oxidative phosphorylation reduce mROS production in direct proportion to the decline in Δψm [53]. In the kidney, mitoHCN3 basal activity has an uncoupling effect and, therefore, a cyto-protective role by attenuating ROS generation.

mitoK channels exposed to ROS produced within the mitochondria can affect their functioning and/or expression [54]. Accordingly, we found that mROS levels were exacerbated by NH_4_Cl-induced acidosis, H_2_O_2_-induced oxidative stress, and ZD7288 after 24 h, causing overexpression of mitoHCN3, which might serve to counteract mROS overproduction. Regulation of mitoHCN3 by oxidative stress was corroborated by mitochondria’s exposure to the free radical scavengers Tiron and NAC. Notably, the full-length mitoHCN3 expression was down-regulated only by Tiron, most likely because Tiron, not NAC, is a mitochondria-targeted antioxidant [55]. These results confirmed inhibition of mitoHCN3 proteolysis by oxidative stress. There are metalloendopeptidases regulated by oxidative stress, such as OMA1 in mitochondria [56] and neprilysin in the apical membrane of the proximal tubules [14]. More studies are necessary to elucidate the regulation of targeted proteolysis of HCN channels by oxidative stress.

Mitochondrial overload with ROS and/or Ca^2+^ contributes to loss of Δψm or depolarization [57]. We reported before that, in HEK293 and H9c2 cells, short-time inhibition of mitoHCN3 (1 h) with ZD7288 causes hyperpolarization by blocking K^+^ influx into the mitochondrial matrix [15,16]. In this work long-time inhibition of mitoHCN (24 h) with ZD7288 produced mitochondrial depolarization only in basal conditions, suggesting a secondary effect of elevated mROS levels [23,51].

Mitochondrial depolarization caused by ZD7288 under basal conditions was insufficient to provoke apoptosis or necrosis, showing that NRK-52E cells could efficiently counteract ZD7288-induced oxidative stress. In this regard, it has been reported that ZD7288 reduces cell death in hippocampal neurons caused by ischemia–reperfusion injury by decreasing the levels of the pro-apoptotic proteins AIF, p53, Bax, and Caspase-3 [22].

In conclusion, our results provide evidence of the participation of mitoHCN3 and lysoHCN3 channels in the control of mitochondrial oxidative stress and autophagy by NH_4_Cl treatment.

## 4. Materials and Methods

### 4.1. Cell Cultures and Experimental Treatments

NRK-52E cells were cultured in high-glucose Dulbecco’s modified Eagle’s medium (DMEM; Gibco, 31600-091, Waltham, MA, USA) supplemented with 10% fetal bovine serum (FBS; Gibco, 26140-079, Waltham, MA, USA) and penicillin (1000 U/mL)/streptomycin (1 mg/mL; Biowest, L0022, Nuaille, France) and maintained in an incubator at 37 °C with a 5% atmosphere of CO_2_. Then, 1–30 mM NH_4_Cl was applied in an FBS-free medium containing 1.5 g/L NaHCO_3_ for 1 or 24 h. Cells were subjected to oxidative stress with 50 µM H_2_O_2_ for 24 h (Sigma, 216763, St. Louis, MO, USA) in a medium supplemented with FBS and 3.7 g/L NaHCO_3_. Antioxidant treatment was performed with 5 mM Tiron (Fluka, 89460, Charlotte, NC, USA) and 10 mM N-acetylcysteine (NAC; Sigma, A7250, St. Louis, MO, USA) for 24 h. HCN channels were blocked with 50 µM ZD7288 for 1 or 24 h (Tocris, 1000, Bristol, UK). Chloroquine (50 µM; Sigma, C6628, St. Louis, MO, USA) and rapamycin (200 nM; Santa Cruz Biotechnology, sc-3504B, Dallas, TX, USA) were used as an inhibitor and an activator of autophagy, respectively.

### 4.2. Mitochondria Enrichment

NRK-52E cells seeded in 15 cm Petri dishes at the end of the treatments were rinsed with cold phosphate-buffered saline (PBS), harvested with 2 mL of PBS, and centrifuged at 600× *g* for 10 min at 4 °C. The pellet was resuspended and homogenized in mitochondria isolation buffer (MIB: 200 mM sucrose, 10 mM Tris/MOPS pH 7.4, 1 mM EGTA) with a glass homogenizer and Teflon pestle attached to a drill; three steps at medium speed were applied. The homogenate was passed through two syringes (18-gauge ½ inch and 27-gauge ½ inch) and centrifuged at 600× *g* for 10 min at 4 °C. The supernatant was transferred to a 1.5 mL tube and centrifuged at 10,000× *g* for 10 min at 4 °C. The mitochondrial pellet was washed with MIB, centrifuged at 10,000× *g* for 10 min at 4 °C, and resuspended in radioimmunoprecipitation assay (RIPA) buffer (50 mM Tris-HCl, pH 7.4; 150 mM NaCl; 1 mM EDTA; 0.5% sodium deoxycholate; 1% Nonidet P-40; 0.1% sodium dodecyl sulfate; 25 mM NaF; 1 mM Na_4_P_2_O_7_; 1 mM Na_3_VO_4_; and 0.5 mM glycerophosphate).

### 4.3. Western Blots

Proteins were separated by sodium dodecyl sulfate-polyacrylamide gel electrophoresis (SDS–PAGE) and electrotransferred to a polyvinylidene fluoride (PVDF) membrane. Membrane was blocked with 5% fat-free milk in Tris-buffered saline supplemented with 0.1% Tween 20 (TBS-T) for 1–2 h at room temperature. Primary antibodies were incubated with the membrane for 18 h at 4 °C: anti-HCN3 (1:400 dilution; C-terminal epitope, Alomone, APC-057, Jerusalem, Israel), anti-Beclin1 (1:1000 dilution, Santa Cruz Biotechnology, sc-48341, Dallas, TX, USA), anti-LC3B (1:250 dilution, Santa Cruz Biotechnology, sc-271625, Dallas, TX, USA), anti-p62 (1:500 dilution, Abnova, H00008878-M01, Neihu District, Taipei City, Taiwán), anti-Parkin (1:300 dilution, Santa Cruz Biotechnology, sc-32282, Dallas, TX, USA), and anti-malondialdehyde (1:500 dilution, Abcam, Ab27642, Waltham, MA, USA). As loading and cell fractionation controls, anti-β-actin (1:1000 dilution, Santa Cruz Biotechnology, sc-47778, Dallas, TX, USA) and anti-VDAC1 (1:3000 dilution, Abcam, Ab34726, Waltham, MA, USA) were used. Secondary antibodies were conjugated to horseradish peroxidase (IgG-HRP, Jackson ImmunoResearch, West Grove, PA, USA) for 1 h at room temperature. Proteins were detected by enhanced chemiluminescence (Clarity Western ECL substrate kit, Bio-Rad, Hercules, CA, USA), and images were obtained with a photo documenter (Analytik Jena, UVP ChemStudio, Upland, CA, USA).

### 4.4. Immunofluorescence Assays

NRK-52E cells were incubated with LysoTracker Red DND-99 [75 nM] for 2 h, a selective lysosome dye (Invitrogen, L7528, Eugene, Oregon USA) and, subsequently, the excess was washed with PBS. The cells were then fixed with 4% paraformaldehyde (10 min), permeabilized with 0.1% Triton X-100 (10 min), and blocked with 2% bovine serum albumin in PBS (1 h). The cells were incubated with rabbit anti-HCN3 (1:100 dilution, Alomone Labs, APC-057, Jerusalem, Israel) overnight at 4 °C, followed by the secondary anti-rabbit IgG antibody coupled to Alexa Fluor 488 (1:500 dilution, 1 h at room temperature, Abcam, Waltham, MA, USA). After washing with Tween 0.5% in PBS, a mounting medium (Vectashield, Vector laboratories, H1000, Newark, CA, USA) was added to the slides. The images were acquired by confocal microscopy (Zeiss LSM 880, Oberkochen, Germany), objective 63x, and analyzed with Fiji ImageJ software v2.7.0.

### 4.5. Measurement of mROS and Δψm

NRK-52E cells were seeded on 96-well plates at a density of 15,000 cells/well. At the end of the treatments, the cells were rinsed with PBS, and the mROS level was measured by incubating the cells with 5 mM MitoSox (excitation: 510 nm, emission: 580 nm; Invitrogen, M36008, Eugene, OR, USA) for 10 min at 37 °C and 5% CO_2_. Nuclear counterstaining was performed with 20 mM Hoechst 33,258 (excitation: 352 nm, emission: 454 nm; Fluka, 14530, Charlotte, NC, USA) for 20 min to normalize the fluorescence signal. The cells were incubated with 7 µmol/mL JC-1 dye (Molecular Probes, T3168, Eugene, OR, USA) for 30 min in a serum-free medium and washed with PBS to remove excess. Incubation with 50 μM carbonyl cyanide m-chlorophenylhydrazone (CCCP; Sigma, C2759, St. Louis, MO, USA) for 10 min was used as a control. Depolarization-related (green) fluorescence was measured at 525 nm, and hyperpolarization-related (red) fluorescence was detected at 590 nm (excitation: 488 nm). The JC-1 signal was calculated as the ratio of red fluorescence to green fluorescence and normalized concerning the control. Data and representative images for both mROS and Δψm assays were acquired with a Cytation 5 Cell Imaging Multi-Mode reader (BioTek Instruments, Inc., Winooski, VT, USA).

### 4.6. Statistical Analysis

Ordinary one-way ANOVA and unpaired Student’s *t*-tests were used to compare the differences between experimental groups. All statistics were carried out using GraphPad Prism v6.01 (Boston, MA, USA).

## Data Availability

All data generated or analyzed during this study are included in this published article.

## Supplementary Materials

The following supporting information can be downloaded at: https://www.mdpi.com/article/10.3390/ijms26189227/s1.
