# Supplementary material for: Inhibition of HCN Channels Enhances Oxidative Stress and Autophagy of NRK-52E Cells Under NH4Cl Treatment
Source: Int J Mol Sci. 2025 Sep 21;26(18):9227. doi: 10.3390/ijms26189227 (PMC12471236; doi:10.3390/ijms26189227)
Supplement: Supplementary file 1 [file ijms-26-09227-s001.zip › Supplementary figure captions.pdf]

Figure S1. ZD7288 or NH_4Cl-induced acidosis does not affect cell viability. (A) Representative dot plots of flow cytometry analysis from NRK-52E cells. Graphs show healthy (Q4), apoptosis (Q3) and necrosis (Q1) cell percentage under basal (CT), NH_4Cl (30 mM) and ZD7288 (50 μM; ZD) conditions for 24 h (n=5). **(B)** Immunoblotting of Apoptosis-Inducing Factor (AIF) from total homogenate of NRK-52E cells (n=3). Densitometric analysis displays the AIF relative abundance normalized with respect to β-Actin (loading control) and the control (CT) group. Data are represented as the mean ± SEM. O*rdinary one-way ANOVA* followed by Newman-Keuls multiple comparisons test as post-hoc test, **p*<0.05.

Figure S2. Overexpression of HCN4 channel in HEK293 cells promotes the expression of LC3βII. **(A)** Representative western blot of HCN4, β-Actin and LC3βII from total homogenates of HEK293 cells transfected with pcDNA3 or pcDNA3/HCN4. **(B)** Quantitative analysis of HCN4 and LC3βII relative abundance (n=3). Data are represented as the mean ± SEM. Two-tailed unpaired Student's *t-test*, *p<0.05, **p<0.01.
