# Supplementary material for: Inhibition of HCN Channels Enhances Oxidative Stress and Autophagy of NRK-52E Cells Under NH4Cl Treatment
Source: Int J Mol Sci. 2025 Sep 21;26(18):9227. doi: 10.3390/ijms26189227 (PMC12471236; doi:10.3390/ijms26189227)
Supplement: Supplementary file 1 [file ijms-26-09227-s001.zip › Supplementary material and methods.pdf]

Supplemental Information

HCN3 potassium channel inhibition enhances oxidative stress and autophagy of proximal tubule cells under NH_4Cl-induced acidosis

Material and methods

Figure S1

Apoptosis detection by flow cytometry

NRK-52E cells were seeded on a 6-well plate at 150,000-200,000 cells/well density. At the end of the treatments, apoptosis was determined with an ApoDETECT staining kit (Invitrogen, 331200) following the manufacturer's instructions. Briefly, the medium and the cells were recovered in a tube and centrifuged at 4,500 × g for 1 min. The cell pellet was rinsed with cold PBS and centrifuged at 4,500 × g for 1 min. The cells were resuspended in binding buffer (ABB; 10 mM HEPES/NaOH, pH 7.4, 140 mM NaCl, 2.5 mM $CaCl_2$) and stained with Annexin V-FITC (1:20 dilution) for 10 min at room temperature. The cells were rinsed, centrifuged at 4,500 × g for 1 min, and resuspended in ABB. Then, 10 µl of propidium iodide (1 µg/ml) was added for 5 min before flow cytometry analysis. Samples were acquired in an Attune flow cytometer (Attune, Applied Biosystems, Life Technologies Corp., CA, USA) and analyzed with FlowJo v.10 software for Windows (Becton, Dickinson and Company, OR, USA).

Figure S2

Cell cultures and plasmid transfection

HEK293 cells were cultured in high-glucose Dulbecco's Modified Eagle Medium (DMEM; Gibco 31600-091) supplemented with 10% fetal bovine serum

(Biowest, S1650), 1% penicillin-streptomycin (Biowest, L0022) and maintained at 37ºC under saturating humidity in 5% CO_2/95% air. HEK293 cells were transiently transfected using Lipofectamine 3000 (ThermoFisher, L3000015) according to the manufacturer's protocol. In brief, 1 µg of each plasmid pcDNA3 or pcDNA3-hHCN4 were diluted in low serum medium Optimem (Gibco 22600-134) and mixed with the transfection reagent Lipofectamine 3000, incubated at room temperature for 10–15 min and added to cultures. Plasmids used were provided by Dr. Luis Vaca from Instituto de Fisiología Celular, UNAM.

Western blotting

After 24 h of transfection, cells were washed with 1x PBS and lysed in RIPA buffer supplemented with protease inhibitors cocktail (cOmplete Mini, Roche, 04693124001). Proteins were separated in 15% polyacrylamide gel and transferred to polyvinylidene fluoride (PVDF) membrane. The membrane was blocked for 1 h at room temperature (RT) in Tris-buffered saline with 0.1% Tween-20 (TBS-T) containing 5% blotting-grade dry milk (Bio-Rad, 1706404). Primary antibodies were diluted in a blocking buffer and incubated overnight at 4 ºC. Secondary antibodies horseradish peroxidase-conjugated were incubated for 1 h at RT. Dilution of primary antibodies against anti-HCN4 rabbit antibody (APC-052, Alomone, Israel), anti-β-Actin mouse antibody (Santa Cruz Biotechnology, sc-47778) and anti-MAPLC3B mouse antibody (Santa Cruz Biotechnology, sc-271625) was 1:400, 1:10,000 and 1:250, respectively. Secondary antibodies used were 1:3,000 anti-rabbit and 1:5,000 anti-mouse (Jackson ImmunoResearch). Immunoreactivity was detected using Clarity Western ECL substrate (Bio-Rad, 170-5060), and images were obtained with a photo documenter (Analytik Jena, UVP ChemStudio, CA, US).
