# Supplementary figures and images for: Inhibition of HCN Channels Enhances Oxidative Stress and Autophagy of NRK-52E Cells Under NH4Cl Treatment
Source: Int J Mol Sci. 2025 Sep 21;26(18):9227. doi: 10.3390/ijms26189227 (PMC12471236; doi:10.3390/ijms26189227)

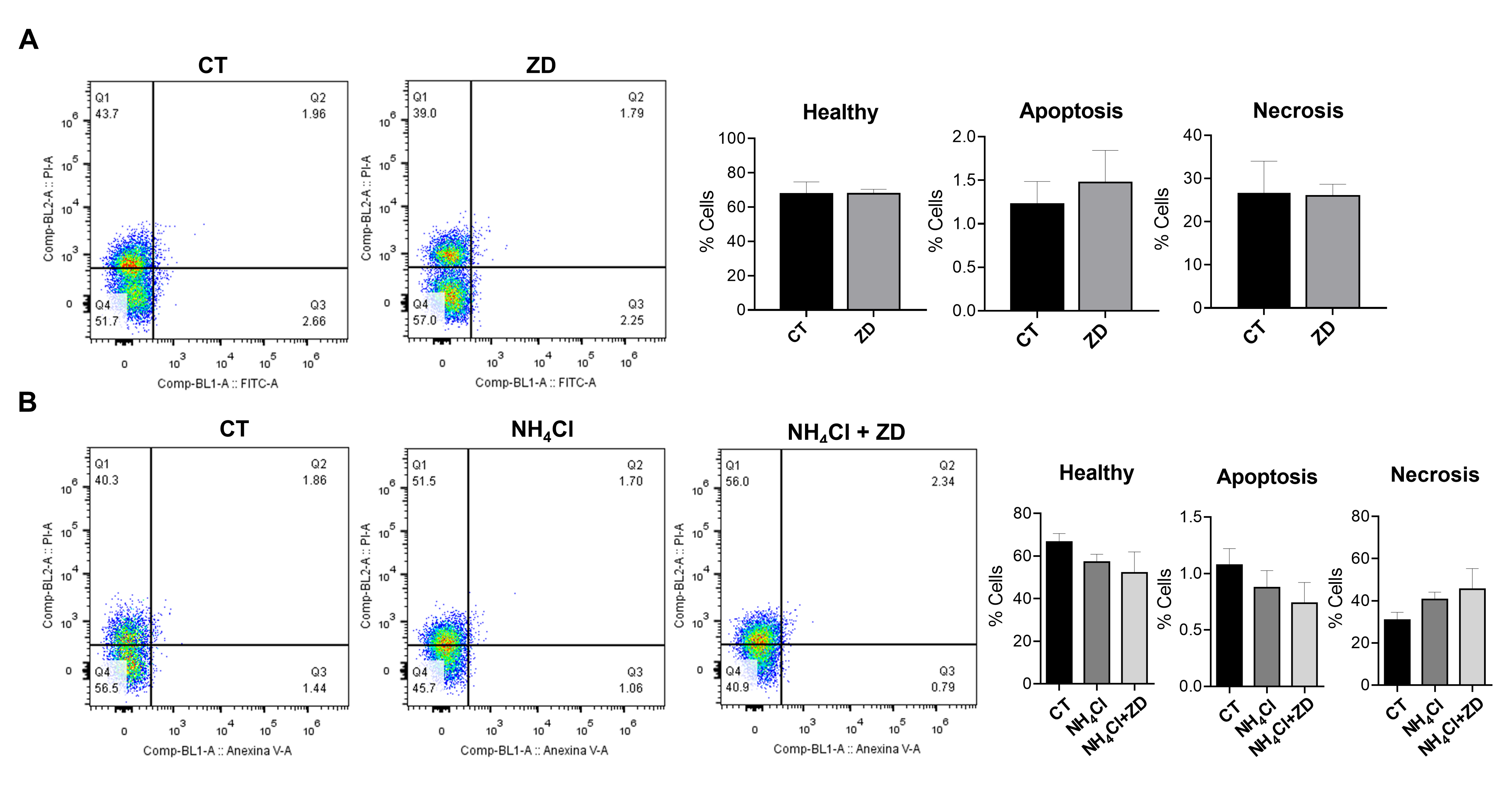

Supplement: Supplementary file 1 [file ijms-26-09227-s001.zip › Figure S1.tif]

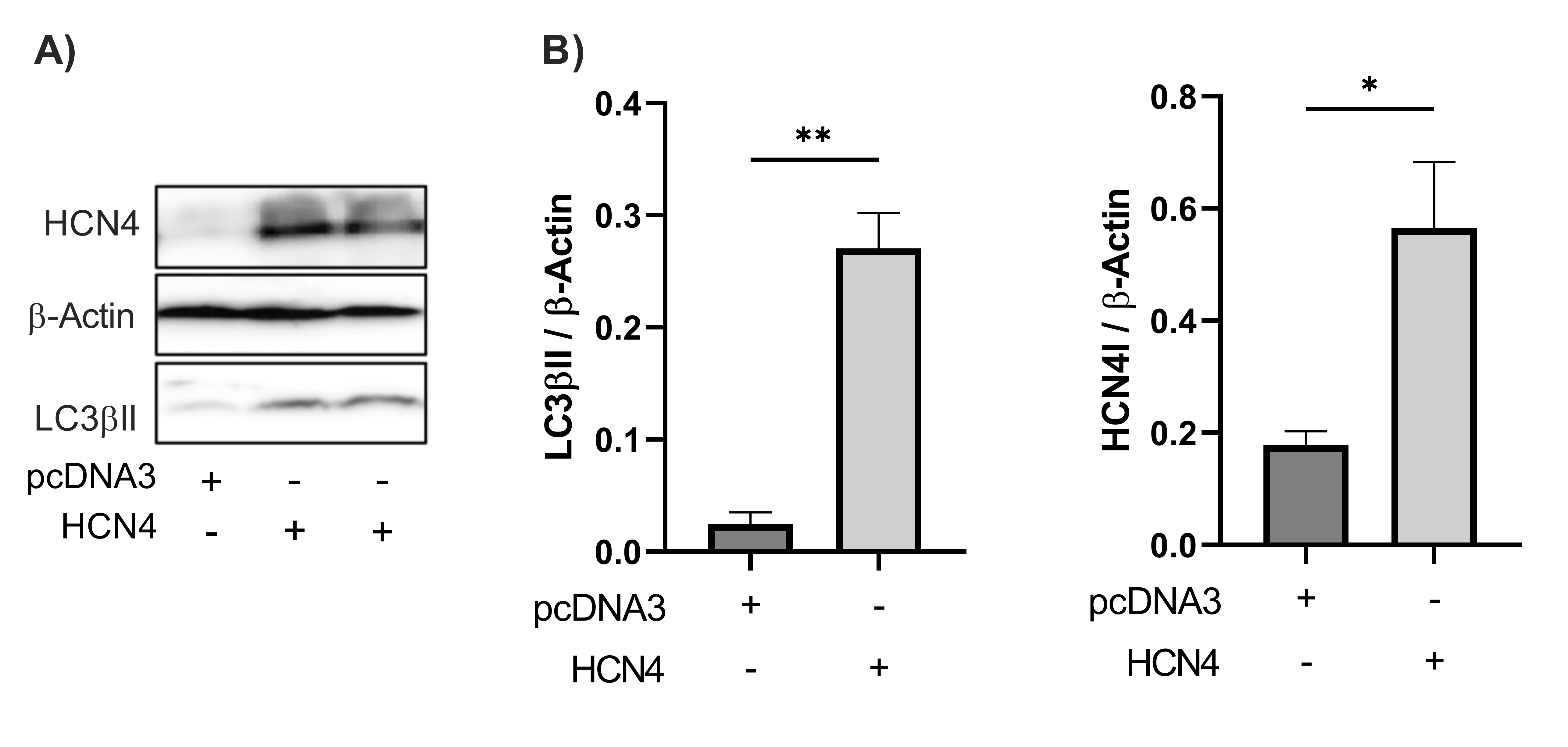

Supplement: Supplementary file 1 [file ijms-26-09227-s001.zip › Figure S2.tif]
